# A case report of a pathology in the ‘whip’ of a flagellicaudatan sauropod

**DOI:** 10.1007/s00114-026-02068-5

**Published:** 2026-02-13

**Authors:** Tom T.P. van der Linden, D. Ray Wilhite, Gunnar T. Bivens, D. Cary Woodruff, Rick J. Hunter, Tim Stecko, Brian D. Curtice

**Affiliations:** 1 Freie Universität Berlin, Berlin, Germany; 2 Het Nationaal Oertijdmuseum, Boxtel, the Netherlands; 3 Auburn University College of Veterinary Medicine, Auburn, AL USA; 4 Mesa Community College, Mesa, AZ USA; 5 Phillip and Patricia Frost Museum of Science, Miami, FL USA; 6 Museum of the Rockies, Bozeman, MT USA; 7 Mountain America Museum of Ancient Life, Lehi, UT USA; 8 North Star Imaging, Orlando, FL USA; 9 Arizona Museum of Natural History, Mesa, AZ USA

**Keywords:** Paleopathology, Flagellicaudata, *Barosaurus*, X-ray computed tomography

## Abstract

Flagellicaudatan sauropods are well known for their elongated tails which end in a ‘whiplash’-like structure, consisting of biconvex rod-like caudal vertebrae lacking a neural arch. A remarkably well-preserved and relatively complete specimen of *Barosaurus* from the Upper Jurassic Morrison Formation preserves a number of these vertebrae, including the last two that terminate the axial series. The penultimate element preserves a distinct callus which we investigated, together with the terminal caudal vertebra, using X-ray computed tomography. A transverse fracture initiated the formation of the callus, highlighted by extensive, disorganized vascularization of the bone outside of the cortex. Healing was likely still ongoing as the vertebra remains in two parts. Although various etiologies can be proposed for the trauma, none can be unambiguously put forward as the cause. The last caudal shows an interesting vascular pattern, and together with the lack of a posterior articular surface, can here be definitely identified as the last vertebra in the axial series. There is substantial variability in the morphology of the last caudal vertebra throughout Sauropoda, and future studies should focus on the soft tissues in this part of the tail, thereby enlightening the potential function of the ‘whip’. We also highlight here the use of high-resolution scanning to reveal the internal structures of fossils in detail when consumptive analysis is not possible.

## Introduction

Paleopathological studies form part of the fundamental basis for studying physiology (e.g., Rothschild et al. 2003; Chinsamy and Tumarkin-Deratzian 2009; Rothschild et al. 2020), life history (e.g., Hanna 2002; Foth et al. 2015), behavior (e.g., Hanna 2002; Baiano et al. 2024), and inter- and intraspecific interactions (e.g., Hanna 2002; Butler et al. 2013; Hone and Tanke 2015) in the fossil record. Due to their informative nature, a general uptake is seen in paleopathological reports in recent years (e.g., Ekhtiari et al. 2020; Gutherz et al. 2020; Hamm et al. 2020; Aureliano et al. 2021; Bertozzo et al. 2021, 2023, 2024; Cruzado-Caballero et al. 2021a, b, 2023; Dyer et al. 2021; Hao et al. 2020, 2021; Słowiak et al. 2021; Arbour et al. 2022; Rothschild et al. 2020, 2022; Woodruff et al. 2022; Anné et al. 2023; Fiorelli and Tykoski 2023; Lei et al. 2023; Smith and Martill 2023; Tan et al. 2023, 2024; Baiano et al. 2024; Samathi et al. 2024; Schaeffer et al. 2024; Xing et al. 2024; Aureliano et al. 2025; Kaikaew et al. 2025; Lacerda et al. 2025; Toefy et al. 2025; Tumanova et al. 2025). Of special interest are healed (i.e., non-lethal) injuries (e.g., Clayton 2018; Hunt et al. 2019; Bertozzo et al. 2021), as they elucidate lifestyle choices of the animal in question, and are occasionally related to inter- and/or intraspecific interactions (such as combat; e.g., Peterson et al. 2013; D’Anastasio et al. 2022).

One non-avian dinosaurian group that has been hypothesized to engage in inter- and intraspecific combat is flagellicaudatan sauropods (Myhrvold and Currie 1997; Tschopp et al. 2015; van der Linden et al. 2025). These sauropods are characterized by extremely elongated tails, consisting of over 80 caudal vertebrae (Gilmore 1936), and are primarily known from the Upper Jurassic (Oxfordian-Tithonian) Morrison Formation of the western United States (van der Linden et al. 2025), though also known from Argentina (Salgado and Bonaparte 1991; Gallina et al. 2014; Gallina 2016), China (Xu et al. 2018), Tanzania (Janensch 1914), Portugal (Bonaparte and Mateus 1999; Mannion et al. 2012), Thailand (Eiamlaor et al. 2025), and possibly India (Bajpaj et al. 2023, but see Mannion and Moore 2025). The last 30 caudal vertebrae of flagellicaudatan tails are characterized by their rod-like morphology, resulting in a ‘whip’-like end of the tail, which appears most extreme in diplodocid flagellicaudatans (Tschopp et al. 2019). The unique tail of flagellicaudatans has been theorized to be used in combat (e.g., Myhrvold and Currie 1997), counterbalancing (Remes et al. 2011), and spatial awareness (Baron 2021). Noise-based signaling (Myhrvold and Currie 1997) through ‘cracking’ (i.e., breaking the sound barrier) of the ‘whip’ was recently challenged by multibody and soft tissue analyses (Conti et al. 2022). The use of the tail in combat situations, either interspecific (predator-prey interactions) or intraspecific, remains plausible, and tail impacts were still considered substantial (Conti et al. 2022).

Sauropod pathologies from the Morrison Formation have been reported in *Camarasaurus* sp. (Tschopp et al. 2014), Diplodocinae indet. (Woodruff et al. 2022), *Apatosaurus* cf. *ajax* (Lovelace 2014), *Haplocanthosaurus* sp. (Tschopp et al. 2019), with tail pathologies having been reported in a variety of taxa (Rothschild and Berman 1991; Rothschild and Martin 2006), and bite marks known from nearly every sauropod taxon (Lei et al. 2023). The aforementioned tail pathologies, however, are all cases of diffuse idiopathic skeletal hyperostosis in mid-caudal vertebrae (following Tschopp et al. 2015), which have been suggested to be indicators for sexual dimorphism (Rothschild and Berman 1991; Rothschild and Martin 2006).

Here, we report, using X-ray computed tomography (CT), on a rather complete specimen of *Barosaurus* sp. from the Upper Jurassic Morrison Formation, which presents a fracture in the penultimate caudal vertebra that partially healed.

### Institutional abbreviations

- AMNH FARB – American Museum of Natural History, New York City, New York, USA. Fossil Amphibian, Reptile, and Bird Collections
- CM – Carnegie Museum of Natural History, Pittsburgh, Pennsylvania, USA
- GMNH – Gunma Museum of Natural History, Gunma, Japan
- LSUSVM – Louisiana State University School of Veterinary Medicine, Baton Rouge, Louisiana, USA
- LPB (FGGUB) – Laboratory of Palaeontology, Faculty of Geology and Geophysics, University of Bucharest, Romania
- NAMAL – Mountain America Museum of Ancient Life, Lehi, Utah, USA
- YPM VP – Yale Peabody Museum, New Haven, Connecticut, USA. Vertebrate Paleontology collection

## Material & methods

The specimen described here (NAMAL-106) was found and excavated from Bone Cabin Quarry in 1995–1996. NAMAL-106 is assigned to *Barosaurus* sp. based on morphological similarities with the holotype (YPM VP.000429) of *Barosaurus lentus* (Marsh 1890; Lull 1919) and the comparable referred specimen AMNH FARB 6341 (McIntosh 2005). The specimen consists of eight cervical, nine dorsal, five sacral, and forty-one caudal vertebrae, including the posteriormost three caudal vertebrae. Cervical and dorsal ribs are preserved, as well as over thirty chevrons. Appendicular elements include a complete scapulocoracoid and most elements of the hindlimbs. A detailed osteological description of NAMAL-106 is in preparation by the authors and thus will not be covered here.

The terminal two caudal vertebrae (CdA and CdB, see below) were imaged with an NSI X3000 industrial CT scanner using a voltage of 190 kV. CT data is available at the following Morphosource link:  https://www.morphosource.org/concern/media/000807553. Segmentation and 3D model generation were performed in Mimics 25.0.1.583 (Materialise, Belgium).

## Results

### Description of the caudal vertebrae (Figs. 1 and 2)

We herein choose to limit the description to the terminal two caudal vertebrae. Because the numerical serial position of these two elements is unknown, as no complete *Barosaurus* tails are known, we choose to refer to them as caudal vertebra (Cd) A and B anteroposteriorly. Measurements of CdA and CdB can be found in Table 1.

CdA is a rod-like, anteroposteriorly elongate vertebra (Fig. 1). No chevron facets or neural arch are present, comparable to other ‘whiplash’ vertebrae (e.g., Holland 1915; Tschopp et al. 2019). The main body is subcircular in cross-section. Towards the articulation surfaces, the centrum expands marginally. The vertebra is biconvex, whereby both articulation surfaces are cleft by a vertical groove, resulting in a dorsal and ventral lobe of the convex articular surface. A distinct expansion is present just posterior to the middle of the vertebra. This expansion is much more prominent compared to the expansion of the centrum body towards the articular surfaces. A break splits this expansion transversely, which is partially filled in with sediment. Based on the internal structure (see below), we confidently conclude that these are not two vertebrae. Inside the break, the bone appears weathered and broken.

**Fig. 2:**
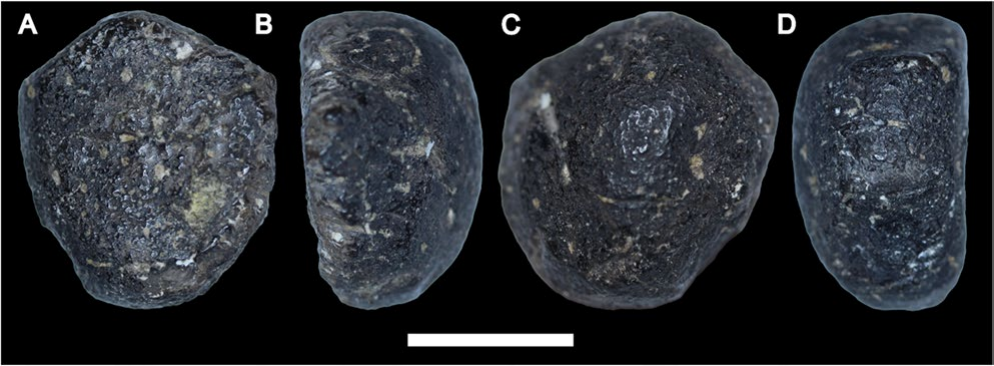
The terminal caudal vertebra (CdB) in anterior (**A**), ?left lateral (**B**), posterior (**C**), and ?right lateral (**D**) views. Scale bar equals 1 cm

CdB is the last caudal vertebra (Fig. 2). The element is subcircular in anterior and posterior view, but unlike the preceding vertebrae, it is not a rod-like element. Instead, the element is a small, knob-like element with a flat anterior articular surface and a convex posterior end. Because the element is relatively smooth and shows no signs of weathering/breaks, we interpret this as the final bony element in the tail articulating with the posterior end of CdA.

**Figure:**
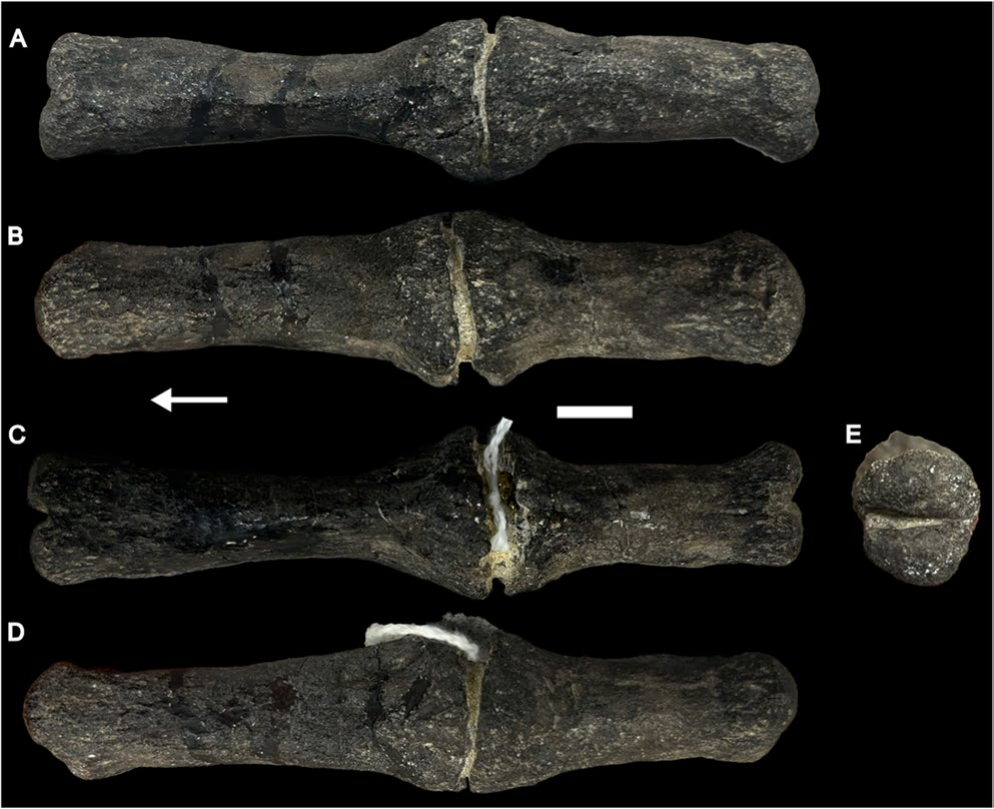
The penultimate caudal vertebra (CdA) in ventral (**A**), left lateral (**B**), dorsal (**C**), right lateral (**D**), and posterior (**E**) view. Arrow points towards anterior. Scale bar equals 1 cm

**Table 1:** Measurements of the posteriormost caudal vertebrae of NAMAL-106. All measurements are provided in mm

	CdA	CdB
Anteroposterior length	107	11
Anterior articulation height	19	18
Anterior articulation width	19	17
Posterior articulation height	20	-
Posterior articulation width	19	-

### Internal structures (Figs. 3, 4 and 5)

**Fig. 3:**
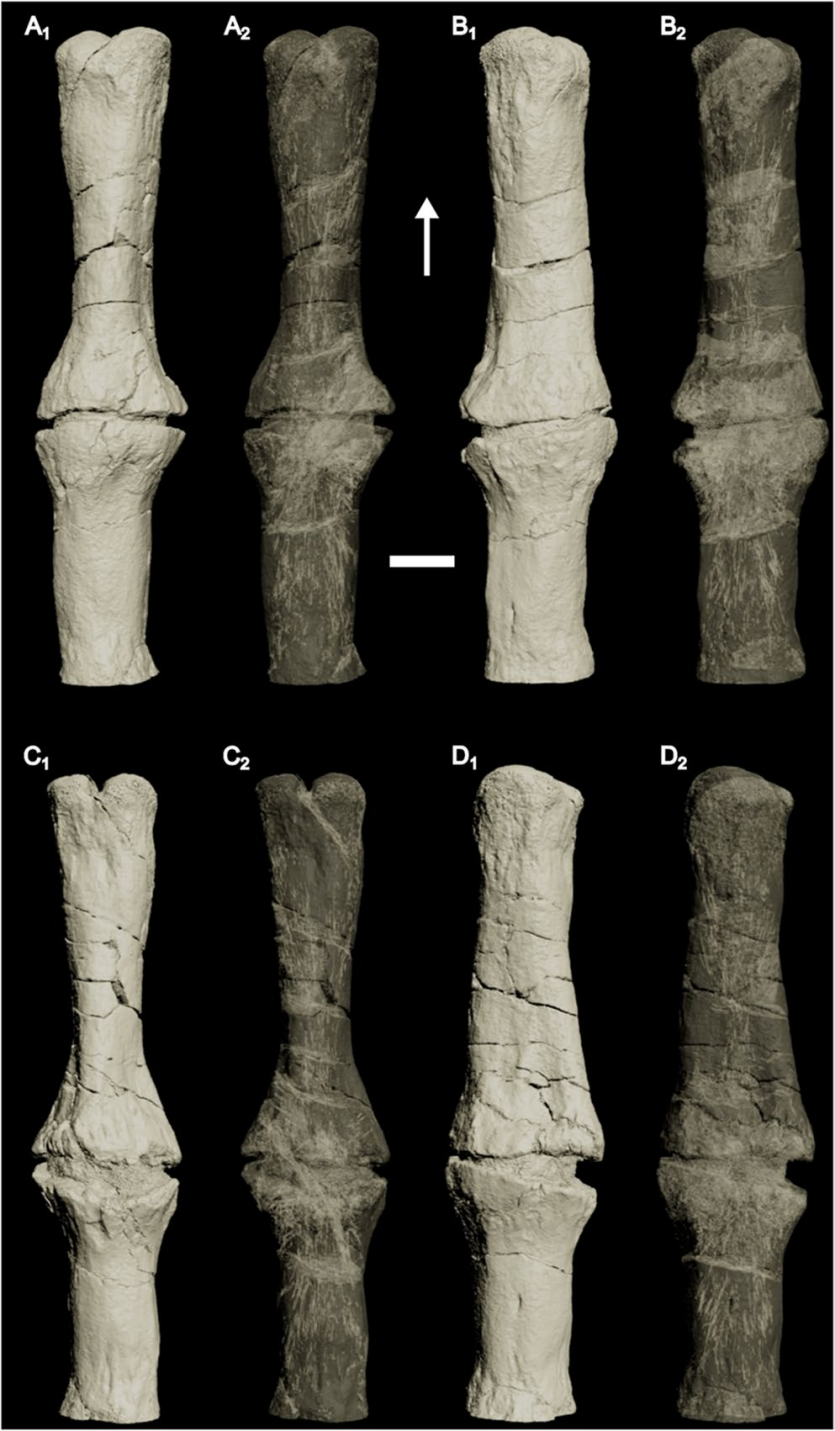
3D model (A_1_, B_1_, C_1_, D_1_) and translucent model (A_2_, B_2_, C_2_, D_2_) of the penultimate caudal vertebra (CdA) in ventral (**A**), right lateral (**B**), dorsal (**C**) and left lateral view (**D**). Note the disorganized vascularization near the callus. Arrow points towards anterior. Scale bar equals 1 cm

**Fig. 4:**
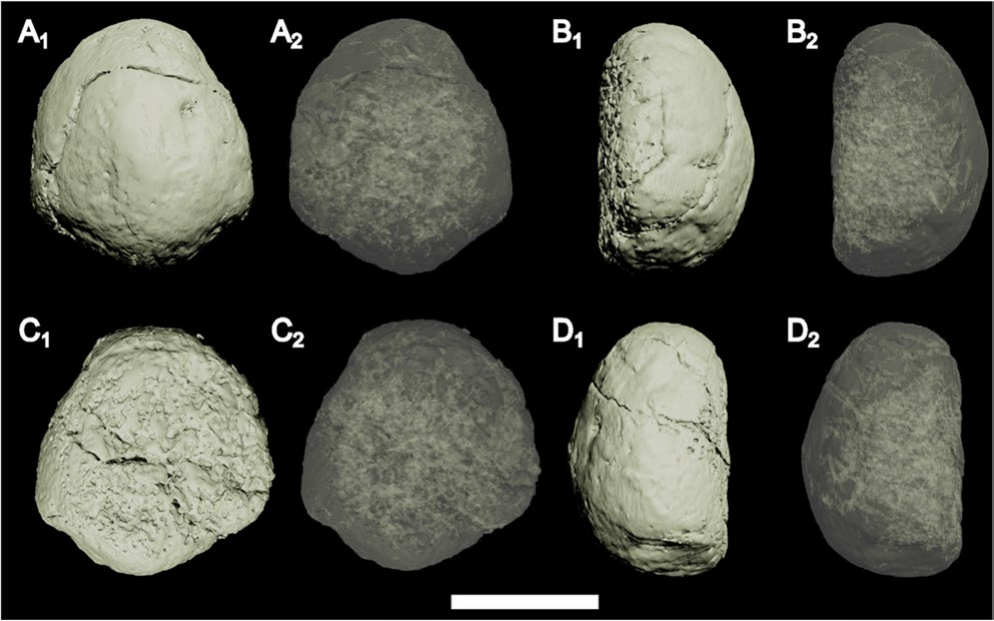
3D model (A_1_, B_1_, C_1_, D_1_) and translucent model (A_2_, B_2_, C_2_, D_2_) of the terminal caudal vertebra (CdB) in posterior (**A**), ?left lateral (**B**), anterior (**C**), and ?right lateral view. Note the origin of vascularization near the articulation surface. Scale bar equals 1 cm

**Fig. 5:**
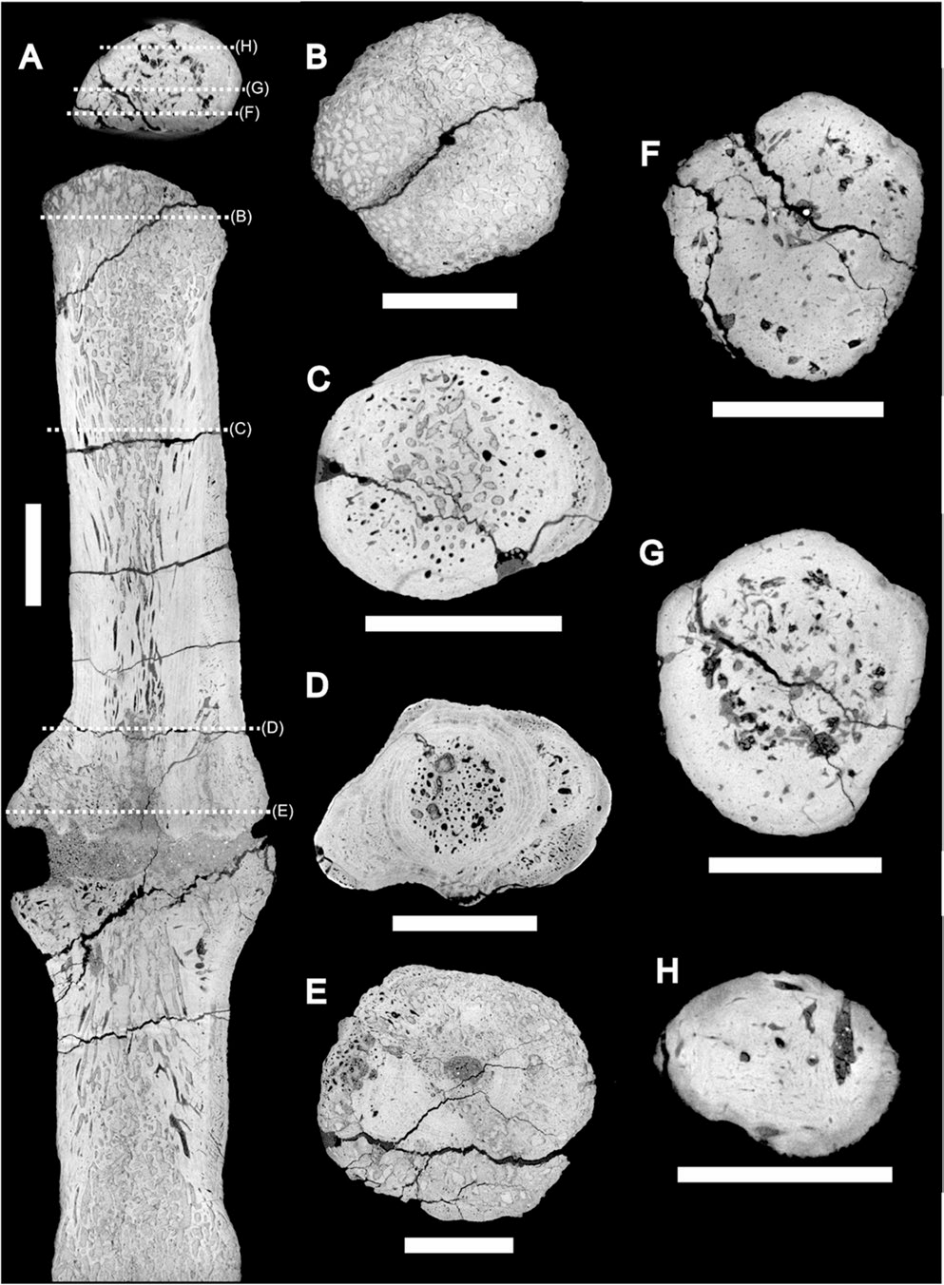
CT sections of CdA and CdB. Sagittal section (**A**) roughly at midheight of CdA and CdB, showing the vascularization and ‘chaotic’ growth near the callus, as well as the sediment dividing the callus. Transverse sections of CdA show the transition from near the anterior articular surface (**B**), normal vertebral bone (**C**), clear periosteal callus formation (**D**), to inside the pathological callus (**E**). Transverse sections of CdB show the transition from near the articular surface (**F**), the middle of the vertebra (**G**), to the posterior rim of CdB. Note that for scanning purposes, CdA and CdB were scanned together, as seen in (**A**). All scale bars equal 1 cm

The anterior and posterior extremities of CdA show clear trabecular bone comprising the articular ends, with very thin cortical bone covering the outer surface (Fig. 5A). Away (i.e., posterior from the anterior surface or anterior from the posterior surface) from the articular surfaces, the trabecular bone becomes ‘denser’ (i.e., less open space), which is laterally encased by cortical bone. Well-defined, numerous anteroposteriorly oriented vascular canals invade the trabecular bone, which makes up the core of the vertebra (Figs. 3 and 5). Within the outer cortex, concentric lineations are present (Fig. 5), which may represent lines of arrested growth. In the middle of the vertebra, the cortical bone laterally expands, and the fracture in between is infilled with sediment. At no point do the bone extremities in the fracture touch each other (Fig. 5A). The vascularization pattern becomes chaotic (Figs. 3 and 5C-D) and is present throughout the entire mediolateral extent of the vertebra near the break and expansion (Figs. 3 and 5D), even outside the concentric lineation. No ‘open’ (i.e., with numerous spaces, as seen at the articulation facet) trabecular bone is present near the fracture (Fig. 5). Internally, CdB consists entirely of cortical bone (Figs. 4 and 5F-H), with a chaotic vascular network originating from the articular surface with which it articulates with the preceding vertebra (Fig. 4).

## Discussion

### Etiologies of the pathology

The pathology, based on macro- and microscopic evidence, can be traced back to a transverse fracture (Lovell 1997), which is related to a traumatic origin, as diseases lead to non-homogeneous calluses (Hao et al. 2021). The periosteally oriented outgrowth forms a distinct callus surrounding the fracture (Chinsamy and Tumarkin-Deratzian 2009), invaded by a disorganized vascular network, distinctly different from the longitudinally aligned vascular canals seen throughout the rest of the vertebra.

However, an exact origin of the pathology is impossible to narrow down. Given the limitations of a single specimen, this fracture cannot be used to distinguish between inter- or intraspecific combat, accidental trauma, or other causes.

At first glance, as previously hypothesized (Myhrvold and Currie 1997; Conti et al. 2022), inter- or intraspecific combat could have caused the fracture. But other origins, such as animal behavior—particularly active movement—generally do not fossilize, so they cannot be excluded. The end of the tail could have (1) hit another object, unrelated to combat, or (2) been hit by another object, be it in active movement (e.g., another object falling on the animal in motion) or in rest (e.g., being stepped on).

An argument possibly against the use of the ‘whip’ in either inter- or intraspecific combat is the lack of pathologies in preserved rod-like vertebrae. If commonly used for defense, it is expected that preserved rod-like vertebrae in flagellicaudatan sauropods exhibit pathologies more often. However, both preservation and soft tissue reconstruction must be considered here. First, this part of the caudal vertebral sequence is rarely preserved, but when present, it is severely understudied. Although several published specimens (Holland 1915; Gilmore 1936; Wilson et al. 1999; You et al. 2003; Tschopp et al. 2019; Mo et al. 2022; Diéz Diáz et al. 2025) do not show evidence of a pathology, not all of these pertain to flagellicaudatan sauropods, such that the sample size of rod-like vertebrae is insufficient to conclude whether pathologies are common in the ‘whip’ of animals like *Barosaurus*.

Secondly, the internal soft tissue surrounding this part of the axial column is unknown in sauropods (Conti et al. 2022), such that we cannot rule out possible protective layering strengthening the ‘whip’ from breaking. In addition, a soft tissue structure protruding posteriorly from the last caudal vertebra could have been present, such as a ‘popper’ (Conti et al. 2022), albeit that there is no evidence from the fossil record for the presence of such a structure. This popper, or an equivalent structure, could have been used to strike instead, such that the caudal vertebrae are less at risk from breakage. Given the limitations of a single specimen, this fracture cannot be used to distinguish between inter- or intraspecific combat, accidental trauma, or other causes. As such, the pathology is neither evidence in favor nor against the hypothesis that flagellicaudatan ‘whips’ were used in combat. We do not rule out any other etiologies.

### The end of a tail

We herein identify CdB as the last caudal vertebra. It was found articulated with the rod-like vertebra, but does not bear its own articular surface on its posterior end, such that no other vertebra can articulate posteriorly with CdB. Preservation of the last caudal vertebra is a rare occurrence in Sauropoda. Confirmed cases include the non-neosauropods *Shunosaurus lii* (Zhang 1988), *Omeisaurus tianfuensis* (Dong et al. 1989), *Mamenchisaurus hochuanensis* (Ye et al. 2001), and *Kotasaurus yamanpalliensis* (Kareem et al. 2024), as all these sauropods are reported to have tail clubs, superficially similar to the ornithischian ankylosaurs (Arbour et al. 2022). These tail clubs only evolved in very selective taxa/clades of the sauropod family tree, as their predecessors, such as *Plateosaurus* (Filek et al. 2025), close relatives, such as *Xinjiangtitan* (Zhang et al. 2020), or the more derived neosauropods (Holland 1906; Gilmore 1925, 1936; Wilson et al. 1999; You et al. 2003; Tschopp et al. 2019) all lack any form of evidence for a tail club. We, however, do not rule out that NAMAL-106, or other neosauropods, evolved soft tissue structures that could be used in an analogous way (i.e., combat/defense) to the tail clubs seen in these early non-neosauropod eusauropods.

In sauropods without tail clubs, only very few specimens (potentially) possess the last caudal vertebra. Among these are the diplodocid *Apatosaurus louisae* CM 3378, stated to be complete by Gilmore (1936), and the immature macronarian *Camarasaurus lentus* CM 11338, of which Gilmore (1925 p. 371) states that “none are missing.” In *Apatosaurus louisae* CM 3378, the posteriormost caudal vertebrae are unfortunately broken (Holland 1915) and crushed. Therefore, the posterior expansion, almost petal-shaped, of the last caudal vertebra may be a taphonomic artifact, and not the ‘true’ end of the tail in terms of vertebrae. It is clear that the last vertebra is still a rod-like element in CM 3378, which is unlike the morphology observed in NAMAL-106, and given this disparate morphology, at this time, we are unconvinced by Gilmore’s (1936) serial positioning of this vertebra. In *Camarasaurus lentus* CM 11338, the tail ends in “a pointed distal caudal” (Gilmore 1925 p. 373), and lacks any resemblance to a rod-like vertebra, which in early diverging macronarians, are far less elongate compared to those of flagellicaudatans (this study; Gilmore 1936) or somphospondylans (Wilson et al. 1999; You et al. 2003). This same pointed caudal morphology can also be seen in *Camarasaurus grandis* GMNH-Pv 101 (McIntosh et al. 1996), and is herein for both *Camarasaurus* specimens interpreted as the terminal caudal vertebra.

The “euhelopodid” — see Moore et al. (2020) for potential taxonomic clarity on the name-bearing *Euhelopus zdanskyi* — *Gobititan shenzhouensis* also likely preserves the last caudal vertebra, but only the distal part of this vertebra is preserved, such that this assignment is based solely on lack of more material from the type locality (You et al. 2003). However, this element is indeed flat, and, albeit crushed, likely not similarly rod-like compared to preceding vertebrae, and therefore here tentatively also interpreted as the last caudal vertebra.

Suteethorn et al. (2009; Fig. 3) indicate in their skeletal reconstruction the presence of the last caudal vertebra in a new specimen of *Phuwiangosaurus*, but there is no mention in the text that indicates the last biconvex rod-like vertebra is indeed the last in the series. An articulated series of rod-like posterior caudals is known from the Maastrichtian of Romania, Lithostrotia *incertae sedis* LPB (FGGUB) R.1568 (Assemblage J, see Diéz Diáz et al. 2025). However, the authors do not clarify whether they interpret the last element as the last caudal vertebra in the series, and it is unclear from Fig. 61 therein or the description whether the last caudal vertebra in the preserved sequence is the last caudal vertebra in the axial series.

A remarkable case of the potential preservation of the last caudal vertebra is *Ruixinia*, which includes an articulated series of 52 caudal vertebrae (Mo et al. 2022). In *Ruixinia*, unlike other titanosauriforms, the last six preserved vertebrae are fused, in a seemingly similar fashion to what is seen in *Mamenchisaurus* (Ye et al. 2001). However, no tail club is formed, unlike what is seen in *Shunosaurus* or *Mamenchisaurus*. The vertebrae remain small, relatively robust rod-like structures. The last vertebra preserved shows a different morphology to the preceding ones, as the posterior surface curves downwards, and thins both transversely and dorsoventrally (Mo et al. 2022; Fig. 8), such that no other vertebra(e) can seemingly articulate with caudal 52. We, therefore, interpret this as the last vertebra in the caudal series for *Ruixinia*.

*Opisthocoelicaudia skarzynskii* may be another sauropod preserving the last caudal vertebra. In the original diagnosis, Borsuk-Bialynicka (1977 p. 8) states, regarding the number of caudal vertebrae: “their number is about 35.” The next page, however, states that “fifty vertebrae are preserved: 11 presacrals, 5 sacrals, and 34 caudals.” No claims are made that the preserved tail is in fact complete, and thus preserves the last caudal vertebra. In addition, the morphology of the last preserved caudal is difficult to assess from the figures in Borsuk-Bialynicka (1977). As the type specimen is currently under redescription (Chiarenza et al. 2025), more osteological information will come forward regarding the completeness of the tail of *Opisthocoelicaudia*.

A similar case of ambiguity is *Rapetosaurus krausei*, wherein the skeletal drawing by Curry-Rogers (2009 Fig. 1) gives some indication of the last caudal being preserved. The last caudal vertebra is not preserved in this taxon with certainty (K. Curry-Rogers pers comm 2025), however, thus precluding comparisons with NAMAL-106.

The current evidence suggests a variety of morphologies terminating the axial series of sauropods, and that the morphology of the last caudal vertebra is significantly different from the penultimate element, as seen in *Crocodilus acutus* (Mook 1921) and *Alligator mississippiensis* (DRW pers obs 2025). In *Crocodilus acutus*, the last caudal vertebra is slightly anteroposteriorly longer than the preceding element, without an articular surface at the posterior end of the last vertebra (Mook 1921). In *Alligator mississippiensis*, the last caudal vertebra transversely thins anteroposteriorly, lacking an articular surface at the distal end for a succeeding caudal vertebra.

The number of caudal vertebrae has been suggested to be inter-, but also intraspecifically variable amongst extant crocodylids (Reese 1915; Mook 1921; Mansfield and Abzhanov 2010), such that caution is warranted with minor variations in caudal count in sauropods, especially in the extremely long tails of flagellicaudatans with their conserved vertebral morphology at the end of the series. Although some variation is likely to be present in the caudal vertebral count, we caution that the amount of variation that can be present. Several vertebral sequences of dissected specimens of *Alligator* (LSUSVM EXAL04,−08, −012) were examined, and the caudal vertebral count was 38, 39, and 38, respectively, with good evidence that both specimens with 38 caudal vertebrae lack the last, 39th vertebra, as both 38th caudal vertebrae show a clear articular surface. However, direct comparisons with flagellicaudatan sauropods are hindered by the different morphology of the caudal vertebrae and the extreme length of the tails in sauropods, with variation in the length of the whip certainly possible (TTPVDL pers obs 2025).

Although differing in morphology, apart from the presence — and to an extent the morphology, see Kareem et al. (2024) — of tail clubs, we do not consider the various morphologies of the last caudal vertebrae between *Barosaurus* (this study) or *Ruixinia* (Mo et al. 2022) to be phylogenetically informative, as significantly more research is still needed to understand the soft tissues (ligaments, tendons, skin) surrounding these rod-like elements, as well as the ontogenetic trajectories of ‘whip’ growth in flagellicaudatan sauropods, given its highly unique morphology amongst sauropod tails.

### CT use in paleopathology studies

The use of X-ray computed tomography in paleontology, ever since its first use in the 1980s, has become ubiquitous in studying the internal structure of fossils (Racicot 2017). In recent paleopathology studies, use of CT scanning is variable, with some only conducting osteohistology (Garros et al. 2025), whereas others apply a combination of osteohistology and CT scanning (Cruzado-Caballero et al. 2021a; Baiano et al. 2024; Bertozzo et al. 2024). Significant advances have been made in recent years in revealing osteohistological information through the use of (µ)CT scanning (Ekhtiari et al. 2020; Hao et al. 2020; Bertozzo et al. 2023; Dreyer et al. 2024), to the point that it can, at smaller scales, replace histological (destructive) sampling (Dreyer et al. 2024; Doneda et al. 2025).

Although osteohistology is an ever-evolving field of study, currently expanding by integration of molecular approaches (Bailleul et al. 2019), (µ)CT can be an excellent alternative to explore histological data, as shown in this study, as well as others (Dreyer et al. 2024; Doneda et al. 2025). Herein, we show that CT scanning can, to an extent, replace ‘traditional’ osteohistological analyses, when circumstances (i.e., the inability for consumptive sampling) warrant a different approach for studying internal structures in fossils.

## Concluding remarks

This is the first report of a pathology in the ‘whip’ part of the tail of flagellicaudatan sauropods, specifically *Barosaurus*. Although the etiology remains uncertain, future investigations of the rarely studied terminus of the caudal sequence in these sauropods may reveal more support for a particular hypothesis presented herein. The preservation of the last caudal vertebra and comparisons with other sauropods raise questions about the soft tissues surrounding the rod-like vertebra. Further support is expressed here for the use of CT scanning to elucidate osteohistological information.

## Data Availability

No datasets were generated or analysed during the current study.
